# Effect of Hyaluronic Acid on the Differentiation of Mesenchymal Stem Cells into Mature Type II Pneumocytes

**DOI:** 10.3390/polym13172928

**Published:** 2021-08-30

**Authors:** Francesca Della Sala, Mario di Gennaro, Gianluca Lista, Francesco Messina, Luigi Ambrosio, Assunta Borzacchiello

**Affiliations:** 1 Institute of Polymers, Composites and Biomaterials, National Research Council, IPCB-CNR (IPCB-CNR), Viale J.F. Kennedy 54, 80125 Naples, Italy; francesca.dellasala@cnr.it (F.D.S.); mariodigennaro5@gmail.com (M.d.G.); luigi.ambrosio@cnr.it (L.A.); 2 Department of Environmental, Biological and Pharmaceutical Sciences and Technologies (DiSTABiF), University of Campania “L. Vanvitelli”, 81100 Caserta, Italy; 3 Neonatologia e Terapia Intensiva Neonatale, Ospedale dei Bambini “Vittore Buzzi”, 20154 Milan, Italy; gianluca.lista@asst-fbf-sacco.it; 4 Ospedale Evangelico Betania, 80147 Naples, Italy; messina52@alice.it

**Keywords:** hyaluronic acid, molecular weights, pulmonary differentiation

## Abstract

Hyaluronic acid (HA) is an essential component of the extracellular matrix (ECM) of the healthy lung, playing an important role in the structure of the alveolar surface stabilizing the surfactant proteins. Alveolar type II (ATII) cells are the fundamental element of the alveolus, specializing in surfactant production. ATII cells represent the main target of lung external lesion and a cornerstone in the repair process of pulmonary damage. In this context, knowledge of the factors influencing mesenchymal stem cell (MSC) differentiation in ATII cells is pivotal in fulfilling therapeutic strategies based on MSCs in lung regenerative medicine. To achieve this goal, the role of HA in promoting the differentiation of MSCs in mature Type II pneumocytes capable of secreting pulmonary surfactant was evaluated. Results demonstrated that HA, at a specific molecular weight can greatly increase the expression of lung surfactant protein, indicating the ability of HA to influence MSC differentiation in ATII cells.

## 1. Introduction

Hyaluronic Acid (HA), a non-sulfated glycosaminoglycan (mucopolysaccharide), composed of repeating disaccharide units of D-glucuronic acid and *N*-acetyl-D-glucosamine linked by *β*-1-3 and *β*-1-4 glycosidic bonds, is present in the connective tissues of all vertebrates. Due to its excellent physicochemical properties such as biodegradability, biocompatibility, and nontoxicity it finds a wide range of applications in the biomedical field such as osteoarthritis surgery, ocular surgery, plastic surgery, tissue engineering, and drug delivery [1]. HA in lung parenchyma is a key component of the ECM and plays the main role in water regulation in the interstitium [2]. HA modulates neutrophil elastase secretion, stimulates or represses immunologic reactions, suppresses bronchial responsiveness, and may play a role in alveolar surface structure stabilizing the surfactant proteins [3]. Lately, in respiratory medicine, HA was successfully evaluated in the treatment of a number of lung diseases in experimental animal models and humans, due to its ability to relieve symptoms. It was determined that the exogenous HA administered by aerosol or tracheal instillation acts against inflammation, protecting the airways against the hyper-reactivity and remodelling of the bronchial and lung parenchyma [4]. For example, in patients affected by chronic lung disorders, repeated administrations of inhaled HA, daily, for eight weeks, induced significant increase in bronchial patency as well as progressive lung deflation with decrease of residual volume [5]. From this perspective, the HA molecule was considered worthy of further exploration for its possible therapeutic role in a variety of respiratory diseases [6]. In particular, lung disorders, such as chronic obstructive pulmonary disease (COPD), idiopathic pulmonary fibrosis (IPF), acute respiratory failure in pneumonia and acute respiratory distress syndrome (ARDS) [7,8,9], and bronchopulmonary dysplasia (BPD) [10], are characterized by derangements of the alveolar walls and altered alveolar functions leading to an impaired oxygen exchange [11]. The main issue is considered the damage of the alveolar epithelial surface, this is composed of two types of epithelial cells (pneumonocytes), type I alveolar (ATI) cells and type II alveolar (ATII) cells [12]. In particular, ATII cells are specialized for the production of surfactant, a complex mixture of proteins and phospholipids, required to reduce the surface tension between alveolar gases and the hydrated epithelial cell surfaces of the alveoli, allowing the respiration process [13,14,15]. ATII cells represent the primary target of external injury in the lung and a key lineage in the repair process critical for reestablishment of normal pulmonary function [16]. In the last decade, new hope for the treatment of lung diseases emerged when several multipotent stem cells populations were found in the lung, a highly quiescent tissue previously thought to have limited reparative capability. For these reasons, increasing attention has been focused on the therapeutic potential of mesenchymal stem cells (MSCs) in repairing damaged lung tissue. As a matter of fact, it has been reported that MSCs possess the ability to differentiate into ATII epithelial cells in vitro [17], and furthermore, MSCs offer modes of action mainly attributed to paracrine mechanisms, with release of pro-angiogenic and anti-apoptotic factors, production of extracellular matrix (ECM), immunomodulators and anti-inflammatory properties. [18]. In this frame, the knowledge of the new factors, influencing the differentiation of MSCs in mature pneumocytes is pivotal for the design of strategies based on stem cell therapy able to restore alveolar damage in lung regenerative medicine. To our knowledge, there are no studies that have explored the role of HA in vitro on the differentiation of pneumocytes. Taking into consideration the HA role in the healthy lung extracellular matrix, the aim of this work was to investigate, for the first time, the effect of HA in promoting the differentiation of MSCs into mature pneumocytes secreting surfactant proteins. Since in the biomedical field, HA applications are related to their physical-chemical properties, such as molecular weight (MW) [19], in this study, the influence of different HA molecular weights, 200 kDa (LMW), 500 kDa (MMW), and 1435 (HMW) on ATII cells differentiation was evaluated. First, HA concentration was optimized by cell viability and viscosity tests and then HA was used to feed umbilical cord mesenchymal stem cells (hUCMSCs). The differentiation of hUCMSCs into ATII cells was evaluated by immunohistochemical analysis and in particular the expression of surfactant protein C (SPC), specific marker of pulmonary differentiation, was investigated along with the other pulmonary surfactant proteins A, B, and D, (SPA, B, and D).

## 2. Materials and Methods

### 2.1. Materials

Hyaluronic acid (HA) with a weight-average molecular weight (MW) of Low (L) 200, Medium (M) 500, and High (H) 1435 kDa were kindly provided by Altergon Italia s.r.l.. Phosphate buffer saline (PBS) tablets without calcium and magnesium were obtained from MP Biomedicals Inc. hUCMSCs cells were extracted from the Wharton jelly of the umbilical cord kindly gifted by Ospedale Evangelico Betania (Naples, Italy), Human Fetal Lung Fibroblast Cells (MRC-5) were purchased from ATCC. Dulbecco’s Modified Eagle Medium (DMEM) (Microgem Italy). Small Airway Epithelial Cell Growth Medium (SAGM) (Lonza C-41 24) and Fetal Bovine Serum (FBS) were purchased from Lonza (Basel, Switzerland). Eagle’s Minimum Essential Medium (EMEM), Hyclone, (MA, USA). Penicillin, streptomycin (10,000 U/mL) from Invitrogen and Life Technologies (Carlsbad, CA, USA) were employed. Trypsin and Ethylenediaminetetraacetic acid (EDTA) were from HiMedia (Mumbai, India). Mitomycin C (MMC), Formalin, bovine serum albumin (BSA) and 4′,6-diamidino-2-phenylindole (DAPI) were purchased from Sigma-Aldrich (USA). Pulmonary-associated surfactant protein C (SPC) rabbit antibody from Abcam. Fitch-conjugated anti-rabbit antibody (Millipore, Billerica, MA, USA). Human pulmonary surfactant associated protein A-B-C and D ELISA kits were obtained from Elabscience.

### 2.2. Hyaluronic Acid Preparation

In order to set the optimum concentration of HA for the pulmonary differentiation experiments, cell viability was performed using L, M, and HMW HA at 0.1%, 0.25%, 0.5%, and 1% (*w*/*v*) respectively. HA was dissolved in DMEM and stirred for 3 h in order to guarantee the homogeneity of the solution. To carry out the differentiation experiments, HA in the form of sodium salt was dissolved in SAGM and then kept with stirring for 3 h. The solutions obtained from the previous procedure were sterilized by filtration using 0.22 micron filters.

### 2.3. Cell Culture

hUCMSCs cells were used at early passages (1–6). The cells were grown in a T-75 cell culture flask (Falcon, Italy), in complete culture medium DMEM, supplemented with 10% FBS and antibiotics (penicillin G sodium 100 U/mL, streptomycin 100 U/mL), in a humidified and controlled atmosphere at 37 °C and 5% CO^2^. The medium was changed every 3–4 days. When confluent growth was reached, the cells were detached with 0.25% trypsin–EDTA solution and washed twice with PBS. The resulting cell suspensions were centrifuged (5 min, 1000 rpm; BRK55/10 Centrifuge by Centurion Scientific Ltd., Chichester, UK), the supernatant separated, and the cells re-suspended in fresh culture medium. Viable cells were counted using the TC20 automated Cell Counter (Biorad, Des Plaines, IL, USA).

In order to generate the feeder layer able to provide the correct microenvironment to promote lung differentiation, we used MRC-5 cell line, derived from normal fetal lung mesenchymal tissue as feeder cells. MRC-5 were grown in a T-75 cell culture flask (Falcon, Italy), in EMEM cell culture medium, supplemented with 10% FBS and antibiotics (penicillin G sodium 100 U/mL, streptomycin 100 U/mL), and 2× Non Essential Amino acids, at 37 °C and 5% CO_2_. Cells were grown up to 80% of confluence in the flask and then were treated with 10 μg/mL of MMC in basal medium for 2 h at 37 °C [20]. MMC interferes with DNA synthesis blocking cell proliferation, avoiding the apoptosis process, so vital cells can be seeded as a feeder layer to promote differentiation. Feeder layer cells were plated at a ratio of 1:10 (number of MSCs to MRC-5 cells) in complete medium. To allow the adhesion overnight, fluoro-dishes were pre-coated with 1% gelatin. Next day, hUCMSCs were plated on feeder layer cells for the differentiation studies.

### 2.4. Cell Viability Test

In order to understand the optimum concentration of HA, cell viability assays were performed at different concentrations of HA reported above. hUCMSC cells were seeded at a density of 10 × 10^3^ cells/mL on 96-wells (World Precision Instruments, Inc., Sarasota, FL, USA). The cells were seeded for each well in triplicate cultured up to 24 h, then Alamar blue assay (AB) was performed by adding AB reagent to the samples (at 10% *v*/*v* with respect to the medium) and incubated at 37 °C for 4 h. The absorbance of the samples was measured using a spectrophotometer plate reader (Multilabel Counter, 1420 Victor, Perkin Elmer) at 570 nm and 600 nm. AB is an indicator dye that incorporates an oxidation–reduction indicator that changes color in response to the chemical reduction in the growth medium, resulting from cell viability. Data are expressed as the percentage difference between treated and control to evaluate the percentage of reduction (Reduction %), which is calculated with the following formula:

(1) $$Reduction\;\left(\%\right)=\frac{\left(O_{2}\times A_{1}\right)-\left(O_{1}\times A_{2}\right)}{\left(O_{2}\times P_{1}\right)-\left(O_{1}\times P_{2}\right)}\times 100$$

where *O*_1_ is the molar extinction coefficient (*E*) of oxidized AB at 570 nm; *O*_2_ is the *E* of oxidized AB at 600 nm; *A*_1_ is the absorbance of test wells at 570 nm; *A*_2_ is the absorbance of test wells at 600 nm; *P*_1_ is the absorbance of the control well at 570 nm; and *P*_2_ is the absorbance of the control well at 600 nm. The percentage of reduction for each sample was normalized to the percentage of reduction for the control to obtain the cell viability percentage [21].

### 2.5. Viscosity Characterization

In order to characterize the viscosity of the HA solution, rheological tests were performed by means of a MARS rheometer™ III, HAAKE™ (TermoFisher Scientific, Waltham, MA, USA). The geometry used was Cone C60/1° CS L. In detail, HA in PBS (1X) solution at the optimized concentration of 0.5%, 0.25%, and 0.1% (*w*/*v*) for LMW, MMW, and HMW respectively, were evaluated. Thus, the non-linear flow properties of the investigated biomaterials were evaluated through steady shear measurements to determinate the viscosity η as a function of shear rate, the so-called flow curves. The tests were performed at room temperature of 25 °C.

### 2.6. ATII Differentiation Studies

In order to assess the differentiation of hUCMSC cells, the qualitative expression of surfactant proteins C (SPC) was evaluated by immunofluorescence using SPC antibody. Then, 1 × 10^4^ hUCMSCs were seeded on a fluoro-dish-35 mm (World Precision Instruments, Inc.), HA solubilized in SAGM was used to feed cells while DMEM and SAGM media were used as control (CTR), cell media were changed every 3 days for 21 days of cell culture with fresh medium. After this time, hUCMSC cells were fixed in 10% Formalin for 1 h, permeabilized with 0.1% Triton X-100, blocked with 1% BSA and incubated with SPC rabbit antibody (Abcam) diluted in 1% BSA at 4 °C overnight. After washing with PBS three times, Fitch-conjugated anti-rabbit antibody (Millipore, Billerica, MA, USA) was added to the cells for 3 h at room temperature. Finally, Cell nuclei were stained with blue DAPI for 10 min at 37 °C. Samples were observed by a confocal microscope system (Leica TCS SP5 MP) with 63X oil immersion objectives. Images were acquired with a resolution of 1024 × 1024 pixel. To determine the quantitative expression of human pulmonary surfactant protein A-B-C and D the supernatants for analysis were collected after 21 days of exposure to the biomaterials, and then analyzed using SP A-B-C and D ELISA kits according to the manufacturer’s protocol.

### 2.7. Statistical Analysis

The results were expressed as mean ± standard deviation (SD). Data analysis was performed using Graphpad^®^ software. The repeated results were compared with the one-way analysis of variance (ANOVA) and a *p* value < 0.001 was considered significant.

## 3. Results and Discussion

### 3.1. Optimization of HA Solution

**Figure 1:**
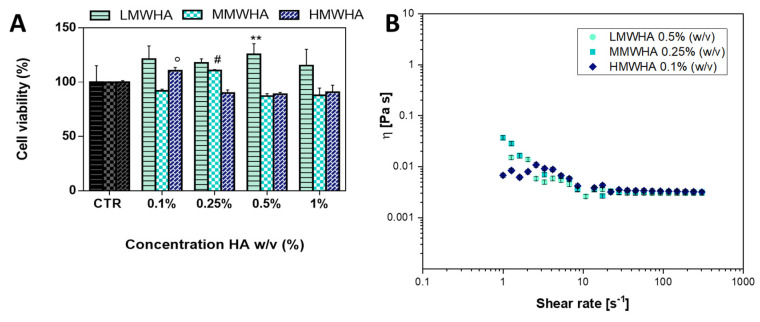
(**A**) Cell viability test after 24 h for hUCMSCs exposed to LMWHA, MMWHA, and HMWHA at concentration of 0.1, 0. 25, 0.5, and 1% (*w*/*v*). All results were presented as mean ± standard deviation. Each error bar represents 1 standard deviation and serves as the estimate of standard uncertainty. The data are representative of 3 repeated experiments in triplicate. ** *p* < 0.01, # *p* < 0.01, ° *p* < 0.01. (**B**) Steady shear tests. Viscosity as a function of shear rate for LMWHA, MMWHA, and HMWHA in PBS solution. The viscosity decreases by increasing the shear rate (shear thinning behaviour).

In order to set the optimized concentration of HA to promote the optimal differentiation, the viability assay was performed. The viability tests were assessed by incubating cells with DMEM cells media implemented with LMWHA (low molecular weight HA), MMWHA (medium molecular weight HA), and HMWHA (high molecular weight HA) at different HA concentrations of 0.1, 0.25, 0.5, and 1% *w*/*v*. As shown from the histograms (Figure 1A), HA was biocompatible, and in particular, the effect of HA can be considered positively on the viability rate of the cells, although some differences can be observed depending on the MW and concentrations of HA. It has been reported that the MW strongly affects the biological function of HA, HMW-HA >500 kDa inhibits cell proliferation, while shorter fragments of HA (i.e., low molecular weight HA (LMW-HA); MW 20–200 kDa) promote cell viability and migration [19,22]. In accordance with the literature, LMWHA was found to have a better effect on cell viability compared the MMWHA and the HMWHA. As regards setting of the HA concentrations to use, we took into consideration the significant concentrations at which the viability exceeded 100% and we set the concentration of 0.5% for the LMWHA, 0.25% for the MMWHA, and 0.1% for the HMWHA. These concentrations, as seen from Figure 1B, have comparable viscosities, and they were optimal for cytocompatibility since solutions at higher viscosities could hamper the passage of nutrients, exert external stress on the cytoskeleton apparatus and thus interfere with cell viability [23,24].

**Figure 2:**
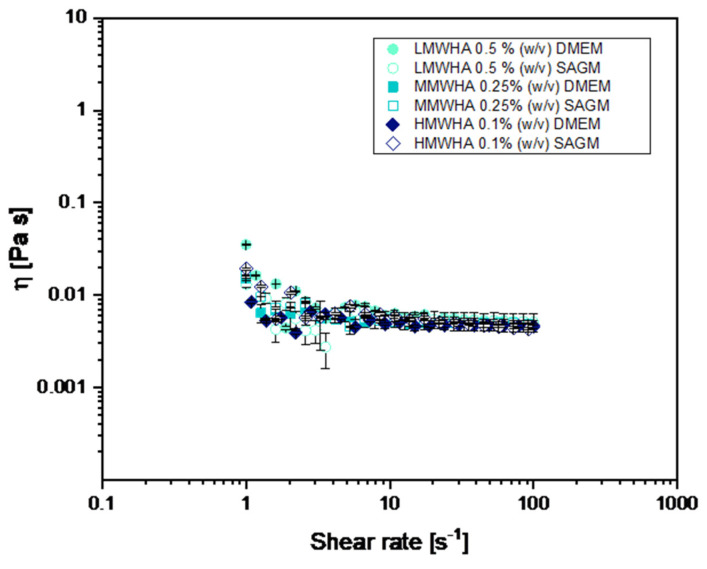
Steady shear tests. Viscosity as a function of shear rate for LMWHA, MMWHA, and HMWHA in DMEM and SAGM cell culture media. The viscosity decreases by increasing the shear rate (shear thinning behaviour).

As can be seen from Figure 1B, the curves for HA at L, M and H MW solubilized in physiological solution showed viscosity curves ranging between about 0.003 and 0.02 Pa s. Moreover, it can be noted that as the HA solutions show a shear thinning behaviour, there is a reduction of the viscosity with increasing rate of shear rate and, in particular, the shear viscosity decreases in an S-shaped fashion. At low shear rate the viscosity is almost constant, decreasing only slightly with the shear rate with a more or less accessible Newtonian plateau. At greater shear rate the viscosity drops sharply with the shear rate (thinning) exhibiting an extended power law region. Increasing further the shear rate, a second Newtonian plateau is present. The shear thinning behaviour can be explained by the dynamics of entanglements and by alignment of the polymer coils. At low shear, the rates of molecular entanglements, such as the time required to a given chain to relax one entanglement and to form the next, are higher than the rate of shear and therefore the average entanglement density is constant, and the polymers coils are in almost imperturbable expanded conformation, resulting in almost constant viscosity. As the shear rate increases, the rate of entanglement disruption becomes predominant and the coils align in the flow direction thus leading to the thinning [25]. The viscosity tests were carried out also for the HA solubilized in the cell medium, DMEM and SAGM, shown in Figure 2. The flow curves of HA in the different medium are similar to the one in PBS indicating that all the HA solutions studied have the same viscosity.

### 3.2. Pneumocyetes Differentiation Study

The capacity of the lungs to repair or to regenerate is determined by key factors. These key factors include the lung stem cells‘ ability to proliferate and differentiate to replace damaged cells or tissues. In view of the relevant role played by ATII cells in the repair process critical for restoration of normal pulmonary function, we focused attention on evaluating the effect of HA on the differentiation of hUCMSCs into Alveolar type II cells, monitoring also the cell viability during the differentiation.

**Figure 3:**
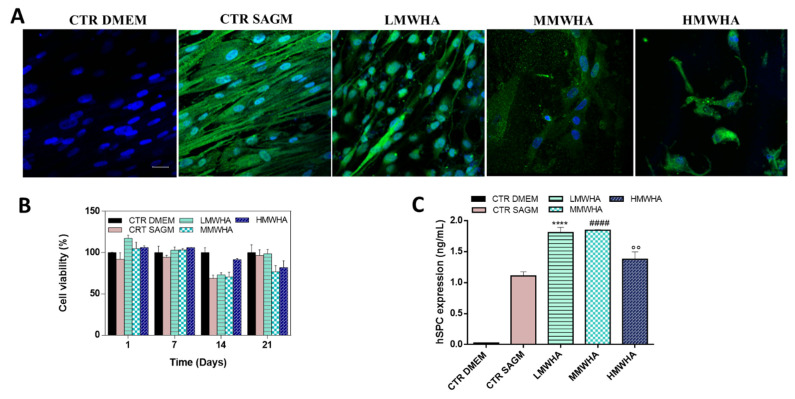
(**A**) Confocal images of Immunofluorescence staining of SPC in differentiated cells. Nuclei were stained with blue DAPI and positive expression of SPC (green) in the cytoplasm were assessed after 21 days of exposure with LMWHA, MMWHA, and HMWHA, excluded for the CTR DMEM. Scale bar: 50 µm (**B**) Cell viability performed by Alamar blue test at 1, 7, 14, and 21 days of culture during the differentiation test (**C**) Quantitative expression of SPC performed by Elisa test. Results are mean ± of 3 experiments. **** *p* < 0.001 vs. Control SAGM, #### *p* < 0.001 vs. Control SAGM, °° *p* < 0.001 vs. Control SAGM.

In order to assess the Alveolar Type II Cells differentiation of hUCMSCs cells, qualitative expression of surfactant protein C (SPC), a specific marker of pulmonary differentiation, was primarily evaluated by the immunofluorescence technique against SPC antibody. The SPC protein is the most important functional protein of the surfactant secreted in the alveoli. In particular, SPC is a trans-membrane protein with a short extra-membrane domain expressed exclusively by alveolar type II cells. It is stored in lamellar bodies with surfactant phospholipids up to secretion in the alveolar space where it increases the stability and diffusion of phospholipids at the air liquid interface thereby promoting the surface tension-lowering properties of the surfactant [26,27]. The confocal images (Figure 3A) show the SPC antibody reactivity after 21 days of incubation with LMWHA, MMWHA, and HMWHA. Immunoreactive SPC cells were observed clearly as green fluorescence spots detected in a homogeneously diffused distribution at cytoplasmic level. Furthermore, in Figure 3A it is possible to observe, that the differentiated cells have a morphology different from the known fibroblast-like morphology of the hUCMSCs, showing an approximately cuboidal shape with more regular dimensions, typical of epithelial cells. The cell viability was evaluated during the differentiation time until 21 days of culture (Figure 3B). As observed, cell viability was maintained optimal in all experimental conditions. It was noted that cell metabolic activity, associated to percentage viability rate, was significantly reduced after 14 days in particular after 21 days of cell culture. More specifically, this occurred in the presence of differentiation stimuli induced by HA. It has been widely reported that with the development of cellular differentiation signals an inhibition of cellular proliferation occurs, with the establishment of a state of cellular quiescence [28,29]. Quantitative expression of SPC was evaluated by Elisa kit. As it can be seen from Figure 3C, the presence of HA implementing SAGM media leads always to an increment of the expression of SPC protein. Compared to exposure with SAGM alone as a control, which showed an SPC expression of approximately 1 ng/mL, the use of HA in particular LMWHA and MMWHA led to an increase in SPC protein expression up to approximately 2 ng/mL, while only a slighter increase in the SPC value also occurred with the HMWHA of about 1.5 ng/mL compared to the use of SAGM alone.

Surfactant is composed of 70–80% phospholipids (dipalmitoylphosphatidylcholine-DPPC), about 10% of proteins (SPA, SPB, SPC, and SPD) and 10% of neutral lipids. SPB and SPC alter lipid packing and spreading and enhance the surface tension lowering activity of the lipids, as well as stabilizing the lipid layers during the respiratory cycle. The surfactant proteins SPA and SPD are larger, relatively abundant, oligomeric proteins that are also synthesized and secreted by type II Alveolar cells. SPD and SPA are structurally related members of the collectin family of C-type mammalian lectins that share distinct collagen like and globular, carbohydrate-binding domains. The most important function of SPD in the lung is as a regulator of pulmonary surfactant lipid level, while SPA is required for the formation of tubular myelin and plays diverse roles in the host-defense functions of the lung with SPD [30].

**Figure 4:**
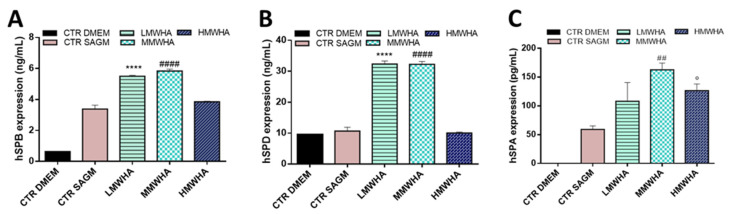
Quantitative expression of SPB (**A**), SPD (**B**) and SPA (**C**) performed by Elisa test. Results are mean ± of 3 experiments. **** *p* < 0.001 vs. Control SAGM, #### *p* < 0.001 vs. Control SAGM, ## *p* < 0.001 vs. Control SAGM, ° *p* < 0.001 vs. Control SAGM.

The expression of the other pulmonary surfactant proteins (SPB, SPD, and SPA) was also evaluated after 21 day of incubation with the HA in order to understand if hUCMSCs are able to differentiate functional ATII cells producing lung surfactant (Figure 4A–C). As shown in the histograms, for SPB the LMWHA and MMWHA improved the expression of around 6 ng/mL compared to the SAGM alone with an expression of about 4 ng/mL. SPD expression was of about 32 ng/mL after exposure of LMWHA and MMWHA, compared to 10 ng/mL expressed with SAGM alone. While for SPA the increment in expression was associated with the exposure of MMWHA with a value of about 152 pg/mL compared to the 50 pg/mL of SAGM alone.

ATII cells, in the wall of the alveolus, are specialized to produce surfactant and they secrete HA into the alveolar aqueous sub-phase. Surfactant is present, on one side with the hydrophobic tails, at the interface with air where it prevents the collapse of the alveolus by lowering surface tension and on the other side with the polar heads in the aqueous sub-phase. This one has enough structure to form a smooth, continuous surface over the epithelial cells and because of its hydrophilic nature it attracts the polar heads of surfactant phospholipids. Surfactant components are packed in the lamellar bodies, which are secreted by ATII cells into the aqueous sub-phase rapidly, in a matter of seconds. During this transit, the lamellar bodies are transformed into tubular myelin, which appears to be the immediate donor of fresh surfactant to the surfactant layer [31]. Indeed, tubular myelin under the surfactant layer makes ridges and grooves in the surfactant layer. These ridges seem to be continuous over long distances and are often concentric as if reflecting some underlying organization. Perhaps, the tubular myelin has lined up on HA molecules and the patterns reflect the configurations that HA is known to take, such as random coil, double helix, hairpin loops, and self-aggregation [32]. It has been recognized that the hydrophobic surfactant proteins, in particular B and C, enhance surface film formation and contribute to alveolar stability. Moreover, it is known that HA in pulmonary healthy ECM (approximately 220 kDa MW) is present in the aqueous sub-phase because ATII cells secrete it [33]. One function of the low molecular weight HA would be to limit the size of any aggregate that HA forms with itself or with proteoglycans in the sub-phase. This would be important since the gel in the sub-phase must be porous enough for the rapid transit of surfactant components. The ability to sequester water, to self-aggregate and to bind many proteins makes HA an ideal molecule to organize a network in the aqueous sub-phase as it is known to fulfil this role in the vitreous humor and in cartilage [34,35,36]. Thus, the primary hypothesis of the role of HA in the lung is that it is present in the aqueous sub-phase of the alveolus and that its function is to create there a hydrophilic gel by self-aggregation and by specific interaction with proteins. The concentration of HA and the other proteins will be greatest at the cell layer and thus the gel will be more structured there, whereas at the air interface it will be mostly water on which the surfactant lipids can spread. A secondary hypothesis, with no analogy in other tissues, is that HA directly interacts with surfactant phospholipids to increase the stability of the surfactant layer. It has been hypothesized that the surfactant proteins, SPB and SPC, in lamellar bodies bind to the hydrophobic regions of the newly synthesized HA and use the HA still bound to the plasma membrane as a trace to move the lamellar bodies into the air interface [37]. Both these two models, the former one hypothesizing a close interaction of HA directly with surfactant proteins thus stabilizing surfactant layers and the latter one hypothesizing that HA is present in the pulmonary sub-phase where it creates a hydrophilic gel network allowing surfactant transit, corroborate our results. HA, thus, represents in vivo a fundamental component of cell microenvironment of the healthy lung ECM and it is recognized that the microenvironment influences cells’ biological behavior [38]. Our results suggested that in vitro HA influenced MSCs differentiation in Type II pneumocytes, these results were confirmed by the enhanced expression of the surfactant proteins SPC, B, D, and A, biochemically identified as specific ATII cells biomarkers [17,39]. In particular, the results here collected indicated that HA, and in specific low and medium MW, could dramatically improve the expression of pulmonary protein surfactant of ATII cells differentiated by hUCMSCs, thus advising an increase of pulmonary surfactant. These findings demonstrated for the first time the ability of HA, in particular low MW, to stimulate and aid the differentiation of hUCMSCs into mature pneumocytes secreting surfactants, which could probably be ascribed to the biomimetic action of HA, physiologically present at a low/medium MW in ECM of lung tissues [11,37]. These outcomes are in agreement with previous literature reporting that HA has a bioactive role in promoting cellular differentiation size-depending based on the different districts of the organism [40]. In vitro tests indicated that HMWHA significantly promoted chondrogenic differentiation of embryo cells or significantly increased the osteogenic differentiation of osteoblast cells compared to LMWHA, since HA macromolecules with these specific MW are present in chrondo and bone tissues [40,41,42]. Currently, the role of HA and the explanation of its mechanism in differentiation of MSCs into ATII is lacking in previous literature. However, it has been reported that MSCs physiologically express the CD44 receptor [43] and this receptor seems to be involved in pulmonary tissue remodeling [44]. It is widely known that many specific biological functions of HA rely on the interaction of HA with its principal receptor CD44. Hence, HA purportedly could exert its biological function in promoting the differentiation of MSCs into ATII cells by interacting directly with these receptors. Moreover, different mechanisms of CD44-HA signaling are discussed in respect to HA MW in order to explain findings of MW dependence in cellular behavior. It has been reported, that LMW HA directly triggers CD44 signal transduction via activation of merlin, PI3K and Erk signaling. Conversely, HMW HA downregulates CD44 signaling mediators [45], and this speculation is in accordance with our results. Anyway, the HA regulatory mechanism underlying the differentiation of MSCs into ATII cells remains to be elucidated and it would be of great interest to investigate it. These results insert a fundamental piece in the knowledge of polymeric substrates that can be used in the stem cell therapy strategies in lung regenerative medicine. In particular, HA macromolecules promoted the differentiation of MSCs compared to the normal medium used to induce in vitro pulmonary differentiation indicating that HA could be further explored for the repair of alveolus damage and in lung regenerative medicine.

## 4. Conclusions

HA is already widely used in clinical practice to relieve the symptoms of lung diseases. In this work, we demonstrated for the first time that HA may play a role in promoting the differentiation of hUCMSCs into mature pneumocytes able to secrete surfactant. The optimal concentrations of HA to feed MSCs to promote differentiation were evaluated by cell viability tests. The viscosity tests suggested that the optimal viability was given by HA solution, at concentrations and molecular weights, having a viscosity in the range of physiological solutions. The study of the differentiation in ATII cells showed that low and medium molecular weight HA was the most suitable in promoting a significant increase in the expression of surfactant proteins, indicating the differentiation in mature pneumocytes. Indeed, HA represents in vivo a fundamental component of the healthy lung ECM. Previous studies have widely demonstrated that HA can have a biological role in helping cellular differentiation depending on its molecular weight in different areas of the organism in which it is present, such as in bone and cartilages tissues. Here for the first time, it was demonstrated that HA at low and medium molecular weight can be used to greatly stimulate the differentiation of MSCs into ATII cells secreting surfactant, mimicking the physiological microenvironment. However, the underlying molecular mechanisms favouring the ATII cells differentiation pathway were not explored in this study. Despite this limitation, which will have to be apprised in the near future, these results represent a first step and a promising breakthrough in the study of HA-based polymeric substrates for lung tissue regeneration.

## Data Availability

Not applicable.
